# Supplementary material for: Agitation near the end of life with dementia: An ethnographic study of care
Source: PLoS One. 2019 Oct 23;14(10):e0224043. doi: 10.1371/journal.pone.0224043 (PMC6808497; doi:10.1371/journal.pone.0224043)
Supplement: Attachment — Submitted filename: Response to Reviewers.docx [file pone.0224043.s001.docx]

| **Comments** | **Response** |
| --- | --- |
| **Reviewer 1** | |
| Greater detail of reporting of the methods, including analysis and data integration is needed. For example, the study is referred to as ethnographic in the title, but there is no definition or even mention of the ethnographic method in the body of the manuscript.  The authors have outlined the three different data collection methods well, but again, these are discrepant from what is referred to in the title.  There is an assertion that innovative and robust triangulation of the data was undertaken (Lines 522 and 586), but there is no description of the data integration/triangulation process in the methods section. The analysis section requires more detail of exactly how the three different data sets were analysed and then integrated/triangulated. | Ethnography is the the study of the culture and social organization of a particular group or community. An ethnographic approach brings in a variety of research techniques as have been used in this study, for example in depth interviews and non-participatory observations. We have added more detail to explain this at lines 119-125 for readers who may not be so cognisant of these research methods. For an example of a classic previous ethnographic work on dying and hospital see Glaser, B,G, and Strauss, A,L, (1965) *Awareness of Dying.* Chicago: Aldine.  We would like to reassure the reviewer that all of the methods we have used are consistent with an ethnographic approach and we have added a paragraph to clarify this (see above). We have also simplified the title.  We give detailed information on how each dataset was analysed in the text:   - Fieldwork observations lines 210-212 - Staff interviews lines 215-218 - Non-participatory structured observations lines 221-226   We have highlighted the section on data synthesis with a sub-heading and expansion in lines 228-238 |
| 2. The results are reported in the wrong order. It would make more sense for the reader (and be more conventional) to report results about the type of staff, then types of behaviours observed, then key themes, then the explanatory model. I suggest that the explanatory model is the result arising from triangulation/integration of the data, hence why it makes more sense for it to be reported as your final interpretation of the multiple results.  Also, the key themes should be named to make it clearer to the reader what they actually were. | We have re-ordered the reporting of the results as requested by the reviewer.  We have added sub-headings |
| 3. There are a number of different terms, concepts and models referred to throughout the manuscript that were not clearly defined and some I suggest unnecessarily complicate the writing and result in a confusing rather than clarifying effect. For example, 'ideal types' is a sociological term borrowed from Weber which has a different meaning to the lay understanding of 'ideal'. As Model 1 clearly is not an ideal way to care for people (according to lay understanding of the word ideal), what is the advantage in using this sociological term? I found the section on 'positional theory' (lines 557-570) to be confusing and nor did I understand its connection to the models you have proposed.  Other key terms/concepts that should be better defined, explained or substituted with simpler terms are 'end of life', 'personhood', 'symbolic interactionist', 'materialistic stance' and 'moral agency'. | We use terms from social sciences and well-established sociological theories (i.e. symbolic interactionism) because this paper is grounded in the social sciences. We have explained and defined concepts in more detail to assist understanding, for example see lines 430-439  We have added explanations for most of these terms. We would not usually explain the terms end of life or personhood in our papers as these terms are widely understood by practitioners and researchers in this field. |
| 4. There was discrepancies in how the observed people with dementia are referred to: at times they are called 'non-participants' and other times they are called 'participants'. Please revise these sections for consistency. | “non-participant” refers to the well-established methodology of non-participant or non-participatory observation, an ethnographic approach where the observer does not interact with the subject. We have edited and now use the term “non-participatory observation” to clarify this. |
| 5. The abstract does not explicitly name the first proposed model, only the second. | This has been corrected |
| 6. Lines 70 - 70 contain some overly long and confusing sentences - suggest revise for improved clarity of meaning. | This has been simplified |
| 7. More detail is needed on the Noticeable Problems Checklist. | Added lines 139-140 |
| 8. It's not clear why you chose to focus only on people with advanced dementia in your study. There is a brief mention of this line 68-9 (and again at 626), but none of the results explicitly refer to scenarios of dying. Greater justification and explanation of the relevance of your study to end of life care is needed. | Dementia takes an unpredictable trajectory and it is difficult to conduct direct observations on people who ae imminently dying. We selected our study population to reflect people with dementia who are at risk of dying within the next 6 months; those in care homes with severe dementia or those in hospitals with moderate or severe dementia and concurrent physical illness. These are “end of life” populations, but care settings do not often recognise this and our paper reflects this reality. We do highlight this in the methods section under “participants”  We have added more justification for this into the introduction and incorporated end of life care more into the discussion. |
| 9. Lines 136-139: were ethical concerns also discussed with site managers? If so, at what point of the study? If not, why not? | This detail has been added line 105-106 |
| 10. Table 1 and 2 - staff and patient demographics should be summarized so as not to identify individuals. E.g. x % male, etc. | We have summarised this data by site (see tables 1). In table 2 we need to give the number of observations and the time spent agitated for each participant, so this table has been partially anonymised. |
| 11. Given that pharmacological intervention for agitated behaviours in dementia are well documented as common clinical practice (although not necessarily best practice), why was there no mention of this type of intervention by staff in your results? | We have added a comment on this in the discussion – lines 636-638 |
| 12. Line 546: Abuse to whom and by whom? | This has been clarified |
| 13. Lines 548-552: I don't believe that this assertion is clearly evidenced by the data presented. | Here we are discussing what is known from the literature, rather than our findings, and so we have clarified the opening to this sentence. |
| 14. Line 622: The term 'moral judgement' differs from the name of model 1, which is 'moral assessment'. Please revise for consistency. | This has been corrected |
| 15. There are a few typographical errors and missing words throughout, including in Appendix 1, which I am sure the authors will identify and correct as part of their revision. | Thank you for highlighting these- we have corrected these issues |
| **Reviewer 2** | |
| 1. On lines 93-7 the authors state that they appointed a personal consultee in order to gain agreement for the participants with dementia to take part in the study. This was because they had been assessed to lack capacity to give informed consent (which would likely be indicated by the severity of dementia of those being recruited), but I wonder if this should be made clear for readers not familiar with the MCA? Likewise a consultee provides advice on what the person’s wishes would have been if they had capacity. The use of the word agreement might suggest some kind of proxy consent, so again just for the purposes of clarity around capacity and consent processes in England and Wales for the wider readership of this journal, I wonder if it would be helpful to also clarify this? Also would it be useful to state the correct technical name for the professional consultee i.e. nominated consultee, given this is provided for personal consultees? | We have added the detail requested to lines 109-114 to make this clear to readers |
| 2. It would be useful to provide details of how the researchers decided when to observe participants in terms of the time periods they spent within each care setting and over what period of time the observations took place if there was more than one for a participant. | This has been added at lines 177-180 |
| 3. In table 3 it would be useful to provide units for the final column, I’m presuming this is total minutes over observation periods-this is not clear. | The units are provided in the title (minutes) |
| 4. On line 249 the authors state patients had a ‘forget-me-not’ magnet on their board. It may be helpful to explain this further for the international readership of this journal who may be unfamiliar with the flower and its meaning in a UK context. | This has been added line 302 |
| 5. It was interesting that in Hospital A, staff predominantly took a MA response to agitated behaviours which was largely a passive rather than active approach, yet the % of the observed period where patients were agitated was the lowest across the four sites. Likewise in site C where staff were most likely to take active and comforting approaches to agitation the percentage of time residents were agitated was the highest. Why might this be? I feel this warrants some further discussion in the context of the participants, models, approaches and institutional contexts within the paper. | This is a very interesting observation however, we need to be careful in how we interpret this data as the % time participants spent agitated at hospital A was 28.3% and at hospital B this was 29.0% so there is only a 0.7% difference between the two sites. The data is only descriptive so we can’t make strong links between these findings. Also, if you look at hospital A, staff only spent 12% of the total time interacting with the agitated patient compared with Hospital B where staff interacted with the patent during 74% of the time when they were agitated |
| 6. The sentence on lines 349-351 needs reviewing. | This has been clarified. |
| 7. Lines 553-4 there is a ) missing here. | This sentence has been amended |
| 8. Lines 560-1 a page number for the quotation is needed | This has been added to the references |
| 9. I would find it helpful if the authors could say more about how positional theory might relate to how moral landscapes of what is seen as permissible or not prevail within organisations and thus how this might be addressed in the case of settings where predominantly ‘chastising’ or ‘looking but not intervening’ approaches are prevalent. There is one sentence that addresses this, but I feel the section would benefit more from discussion of this. | We have added further comment on this into the discussion. |
| 10. Line 603 uses different terms of the models to those given earlier in the paper. It might be helpful to stick to the same terms throughout? | This has been corrected |
| 11. The para lines 621-626 discusses the need to try and move people to a more needs-based model of agitated behaviour assessment, however, given the context that higher levels of agitation were displayed in the settings where staff provided more comforting responses, there is a potential implication that by doing so staff may not in fact reduce agitation levels and thus the burnout risk could potentially be higher. Thus on further implication might be that any movement of staff perceptions will need to be accompanied by interventions that can potentially equip staff with a greater range of skills and resources to help prevent and reduce agitation. | Please see the response to point 5 (above) |
