# Supplementary material for: Agitation near the end of life with dementia: An ethnographic study of care
Source: PLoS One. 2019 Oct 23;14(10):e0224043. doi: 10.1371/journal.pone.0224043 (PMC6808497; doi:10.1371/journal.pone.0224043)
Supplement: Attachment — Submitted filename: Response to reviewer comments R2.docx [file pone.0224043.s002.docx]

**PONE-D-19-18618R Agitation near end of life with dementia: an ethnographic study of care: response to reviewer’s comments**

| Qualitative and quantitative data analysis and integration: The reporting of analysis methods is insufficient. For example, Excel, NVivo and STAT are tools to help manage and store data, not data analysis per se. Please report the exact analysis methods. Line 239 state you used graphs, networks and diagrams to synthesis the data, but none of these are present in the report.    In addition, the authors response to this item does not contain the correct line numbers for the sections referred to. | We have added more detailed information on this process in lines 238-278. The diagrams etc. were working documents to assist with data synthesis and not designed to be presented as “results”.  We apologise. For this response (R2) we have checked line references. |
| --- | --- |
| Table 1, Site B, Role column has '1 senior nurse' listed twice. | This has been corrected |
| Table 2 data requires further summarisation e.g. mean age, total number of observations per site, total time spent agitated per site. | We have added summaries per site and an overall summary for all sites at the bottom of the table |
| Table 3 data simply does not make sense, as % do not add up to 100% either horizontally or vertically. There is an error (% written twice) in the header of column | Row percentages do not add up to 100 as some types of responses occurred simultaneously, for example speech and tactile comfort and so these were double-coded. To explain this we have:   1) added an extra column “% of observed time where participants were agitated, and staff did not respond” (5^th^ column from left, lines 481-482) 2) added information on this in the methods (line 217-218)   The extra % has been removed (line 481) |
| 4. The 'Response to agitation' theme was positioned as reporting staff interview data and yet reports observational data as evidence, which in addition does not identify who the person is whose words are quoted. | This has occurred because of the restructuring of the paper requested at the first review stage. We have moved this section and integrated it with the observational section line 468-476 |
| 1. The term 'non-participant observations' continues to be confusing as it is often used in the same sentence as 'participants'. While it may be a common term in ethnographic research, I suggest that 'structured observation' would be sufficient and also cause less cognitive dissonance from the point of view of the reader. | We have replaced the ethnographic terminology with “structured observations” as requested by the reviewer |
| 2. Lines 80-81: what are the exact prevalence of agitated behaviours in people with more advanced dementia (i.e. needs more specific detail that simply 'common'). | It is not possible to give an “exact” prevalence as this depends on the how agitation is measured so we have provided a range and a comment on severity at lines 80-83 |
| 3. My previous point about sharing ethical concerns with managers referred to concerns that arose during the study, not potential ones. What was the process for reporting back to managers issues of concern witnessed by the researchers? | We have added detail regarding this at lines 129-142 |
| 4. Objectives are stated for the observation aspect of the study, but not for the interviews. | This has been added (lines 159-160) |
| 5. Many punctuation, spelling, grammatical and typographical errors remain. For example, there is use of both American and UK English spelling, use of '&' rather than 'and', multiple spacings between words, and missing or misplaced commas. | We have ensured consistent use of American English all the way through- see track changes version of the text  There was one use of “&” which has been removed from the text and replaced with “and”  Spacings and commas have been checked and corrected (see track changes version of the text |
